# Phosphine Resistance in the Rust Red Flour Beetle, *Tribolium castaneum* (Coleoptera: Tenebrionidae): Inheritance, Gene Interactions and Fitness Costs

**DOI:** 10.1371/journal.pone.0031582

**Published:** 2012-02-21

**Authors:** Rajeswaran Jagadeesan, Patrick J. Collins, Gregory J. Daglish, Paul R. Ebert, David I. Schlipalius

**Affiliations:** 1 School of Biological Sciences, University of Queensland, St. Lucia, Australia; 2 Department of Employment, Economic Development and Innovation, Ecosciences Precinct, Agri-Science Queensland, Brisbane, Australia; 3 Cooperative Research Centre for National Plant Biosecurity, Bruce, Australia; United States Department of Agriculture, Agriculture Research Service, United States of America

## Abstract

The recent emergence of heritable high level resistance to phosphine in stored grain pests is a serious concern among major grain growing countries around the world. Here we describe the genetics of phosphine resistance in the rust red flour beetle *Tribolium castaneum* (Herbst), a pest of stored grain as well as a genetic model organism. We investigated three field collected strains of *T. castaneum viz.*, susceptible (QTC4), weakly resistant (QTC1012) and strongly resistant (QTC931) to phosphine. The dose-mortality responses of their test- and inter-cross progeny revealed that most resistance was conferred by a single major resistance gene in the weakly (3.2×) resistant strain. This gene was also found in the strongly resistant (431×) strain, together with a second major resistance gene and additional minor factors. The second major gene by itself confers only 12–20× resistance, suggesting that a strong synergistic epistatic interaction between the genes is responsible for the high level of resistance (431×) observed in the strongly resistant strain. Phosphine resistance is not sex linked and is inherited as an incompletely recessive, autosomal trait. The analysis of the phenotypic fitness response of a population derived from a single pair inter-strain cross between the susceptible and strongly resistant strains indicated the changes in the level of response in the strong resistance phenotype; however this effect was not consistent and apparently masked by the genetic background of the weakly resistant strain. The results from this work will inform phosphine resistance management strategies and provide a basis for the identification of the resistance genes.

## Introduction

The rust red flour beetle, *Tribolium castaneum* (Herbst) is a serious, cosmopolitan pest of stored grains and grain products in tropical and subtropical regions of the world [1]. Currently, fumigation with phosphine is the major means of control of this species world-wide. Reliance on phosphine is expected to continue for the foreseeable future because of international regulatory and market acceptance of this material and the lack of viable alternatives. A consequence of the heavy use of phosphine has been the development of resistance in several pest species including *T. castaneum* in many regions [2], [3], [4], [5], [6], [7], [8], [9] of the world and this is a major threat to the continued and effective use of this fumigant for the protection of grain and other commodities.

The unique status of phosphine requires that strategies have to be implemented to limit the development of resistance so that use of this valuable fumigant can continue. The foundation of any effective resistance management strategy is an understanding of the processes involved in selection for resistance. Key factors in the rate of evolution of resistance include the number and mode of inheritance of resistance genes, their relative dominance and pleiotropic effects, especially any change in fitness of individuals [10]. The inheritance of resistance to phosphine in *T. castaneum* has been examined using classical methods in three strains with low level resistance, from the Ivory Coast [11], Pakistan [12], and Australia [13], and in one strain originating from Brazil with relatively high level resistance [3]. There was agreement that low level resistance was controlled by autosomal factors and was semi-dominant. However, in the strains from Ivory Coast and Pakistan, phosphine resistance appeared to be controlled by a single gene whereas an additional gene of minor effect contributed to resistance in the strain from Australia. In the single study of high level resistance, it was concluded that resistance was controlled by two recessive genes [3]. There is no information regarding relative fitness of weakly and strongly resistant strains.

Both high and low level phosphine resistance phenotypes have now been detected in population samples of *T. castaneum* in Australia (Collins PJ, unpublished). As a contribution to the development of sustainable management of phosphine resistance in this species, we conducted a number of genetic experiments with several field collected resistant strains that are homozygous for weak and strong resistance traits to determine inheritance patterns, dominance of resistance alleles and any change in fitness associated with resistance. Our working hypothesis was that as the phenotypic response to phosphine in *T. castaneum* is similar to that in *Rhyzopertha dominica* (Linneaus), i.e. two distinct levels of resistance labelled Weak and Strong [14], then the genetic basis of resistance may be similar [15].

## Materials and Methods

### Insect strains: origins and culturing

Two resistance phenotypes have been recognised in *T. castaneum* from Australia, weak-resistance and strong-resistance. The strains used in this study included phosphine susceptible QTC4, designated as S-strain in this report; QTC1012 and QTC1389 both expressing the weak resistance phenotype and designated as Weak-R_1_ and Weak-R_2_, respectively; and QTC931 expressing the strong-resistance phenotype and designated as Strong-R. The S-strain was derived from adults collected from a storage facility in Brisbane, southeast Queensland in 1965 [13] and has been cultured in the laboratory without exposure to phosphine or other insecticides since that time. Weak-R_1_ and Weak-R_2_ were derived from adults collected from small farm storages at Yellarbon in 2001 and Moura in 2006, in southeast Queensland, Australia, respectively. Strong-R was derived from adults collected from a central storage at Natcha, southeast Queensland, Australia, in 2000. The insects were cultured in whole wheat flour and yeast 20∶1 and maintained at 30°C and 55% relative humidity (RH). Before the commencement of genetic crosses, the parental phosphine-resistant strains were maintained under artificial selection for phosphine resistance for five generations to promote homozygosity within the strains.

### Phosphine susceptibility tests

Phosphine was generated and its concentration was determined according to Daglish *et al.* [16]. The mortality of insects due to phosphine exposure was tested according to the FAO standard bioassay procedure [17] using a range of phosphine concentrations (0.005 to 16 mg litre^−1^). Briefly, adult beetles one to three week post-eclosion were fumigated for 20 hours at 25°C and 70% RH. During fumigation, insects were in ventilated plastic vials without food inside gas-tight chambers of fixed volume (4 to 6 litre) into which phosphine had been injected. Mortality was assessed following a recovery period of seven days in whole wheat flour at 25°C and 55% RH.

### Mass inter-strain genetic crosses

To determine the mode of inheritance of the phosphine resistance trait in *T. castaneum* four Mass Inter-strain Crosses (MIC) were set up: S-strain X Weak-R_1_ (MIC); S-strain X Strong-R (MIC); Weak-R_1_ X Strong-R (MIC); and Weak-R_1_ X Weak-R_2_ (MIC). From these crosses, F_1_, F_2_ (inter-cross) and reciprocal F_1_-BC (testcross) progeny were produced. Each cross employed 50 males of one strain and 50 females of the other strain. To account for the possibility of sex-specific inheritance, reciprocal crosses were made. The resulting F_1_ beetles were also used to produce F_2_ inter-cross and F_1_-BC (with the recessive resistant parent i.e test cross) populations. F_2_ insects were obtained by allowing F_1_ progeny to randomly mate with each other for two weeks. F_1_-BC progeny were obtained by identifying approximately 50 F_1_ female insects at the pupal stage and mating these virgin females with approximately 50 males from the resistant parental strain. A reciprocal testcross using 50 males from the F_1_ mated to 50 virgin females of the resistant strain was also performed.

### Single pair inter-strain genetic crosses

Analysis of fitness effects associated with phosphine resistance in *T. castaneum* relied on three Single pair Inter-strain Crosses (SIC): S-strain X Weak-R_1_ (SIC); S-strain X Strong-R (SIC); and Weak-R_1_ X Strong-R (SIC). For each SIC, two-week old virgin adults were paired (one male+one female) and kept on whole wheat flour with yeast (20∶1) for two weeks. The parental insects were then transferred to fresh food and the resulting F_1_ progeny were left on flour for three weeks to allow them to mature to adulthood. The single pair crossing procedure was repeated with F_1_ hybrid insects to obtain F_2_ populations. Two weeks after eclosion, approximately 100 F_2_ adults were transferred to fresh flour to produce an F_3_ generation. The procedure was repeated to the F_20_ generation taking care to prevent mixing between generations.

### Data analysis

#### Mode of inheritance of resistance

Probit analysis using log-concentration-probit mortality (lc-pm) regression [18] was carried out using the Genstat 9.0 statistical package [19]. Mortality response data were corrected using Abbott's formula [20] to eliminate the influence of control mortality, which was not greater than 10% in these experiments. From the regression analysis, the relative potency, LC_50_ [lethal concentration] values and their 95% fiducial limits of reciprocal F_1_ crosses were calculated and used to determine sex-linkage. The degree of dominance was estimated on the basis of dose responses of the F_1_ progeny from reciprocal crosses according to the method of Stone [21]. The resistance ratios were calculated by dividing the LC_50_ values of the resistant parent or the F_1_ hybrid by the LC_50_ of the susceptible strain.

#### Number of genes conferring resistance

Two approaches were used to examine the number of genes conferring resistance. The first approach used the observed response curves of the F_2_ (MIC) and F_1_-BC (MIC) progeny to a range of concentrations of phosphine to estimate the number of genes responsible for resistance. According to Tsukamoto [22] if log concentration–probit mortality (lc-pm) lines of the resistant strain, susceptible strain and their reciprocal F_1_ progenies did not overlap and where a single recessive gene was conferring resistance, then a plateau or point of inflection would occur in the log dose response curve of the F_2_ at around 75% and in the log dose response curve of an F_1_–BC (test cross) at around 50% [23]. The second approach used chi-square goodness-of-fit [24] test to compare observed and theoretical expected mortality values at each concentration, average across the overall curves [25] and the null hypothesis of monogenic inheritance was tested using modified chi-square analyses accommodating heterogeneity factor. The heterogeneity factor was determined as the weighted mean of the individual heterogeneity factors from probit analysis of data from contributing strains [26]. For analyses where the expected response was less than one, the number of observed responses were combined with the value for an adjacent dose and the analyses were adjusted accordingly. The null hypothesis test was rejected when the observed and expected mortality significantly differed (*P*<0.05) after Bonferroni adjustment for multiple comparisions [27].

#### Fitness cost

For fitness cost analysis, we measured if there were any changes to the phenotype in populations with a segregating genotype. The response to phosphine at a range of concentrations (0.0005–12 mg litre^−1^) was measured in generations F_5_, F_10_, F_15_ and F_20_ of each of the three single pair inter-strain crosses, S-strain X Weak-R_1_ (SIC); S-strain X Strong-R (SIC); and Weak-R_1_ X Strong-R (SIC). To identify shifts in phenotype, these data were analysed using a logistic standard ‘s’ curve model with Genstat 9.0 software [19]. For each cross, a grouped regression analysis of the data for each generation tested (F_5_, F_10_, F_15_ and F_20_) was done to determine whether model parameters (linear and non linear) were common across the generations or whether separate curves with independent parameters were the most appropriate to describe the data. The LC_50_ values were calculated and compared for each generation using the standard curve equation; Mortality (Y) = A+C/(1+e ^(−B*(X-M))^), where X is the log dose and B, M (non -linear) and C, A (linear) are the model parameters.

## Results

### Inheritance of weak resistance to phosphine (MIC: S-strain X Weak-R_1_)

#### Resistance levels, maternal effects and degree of dominance

**Figure 1:**
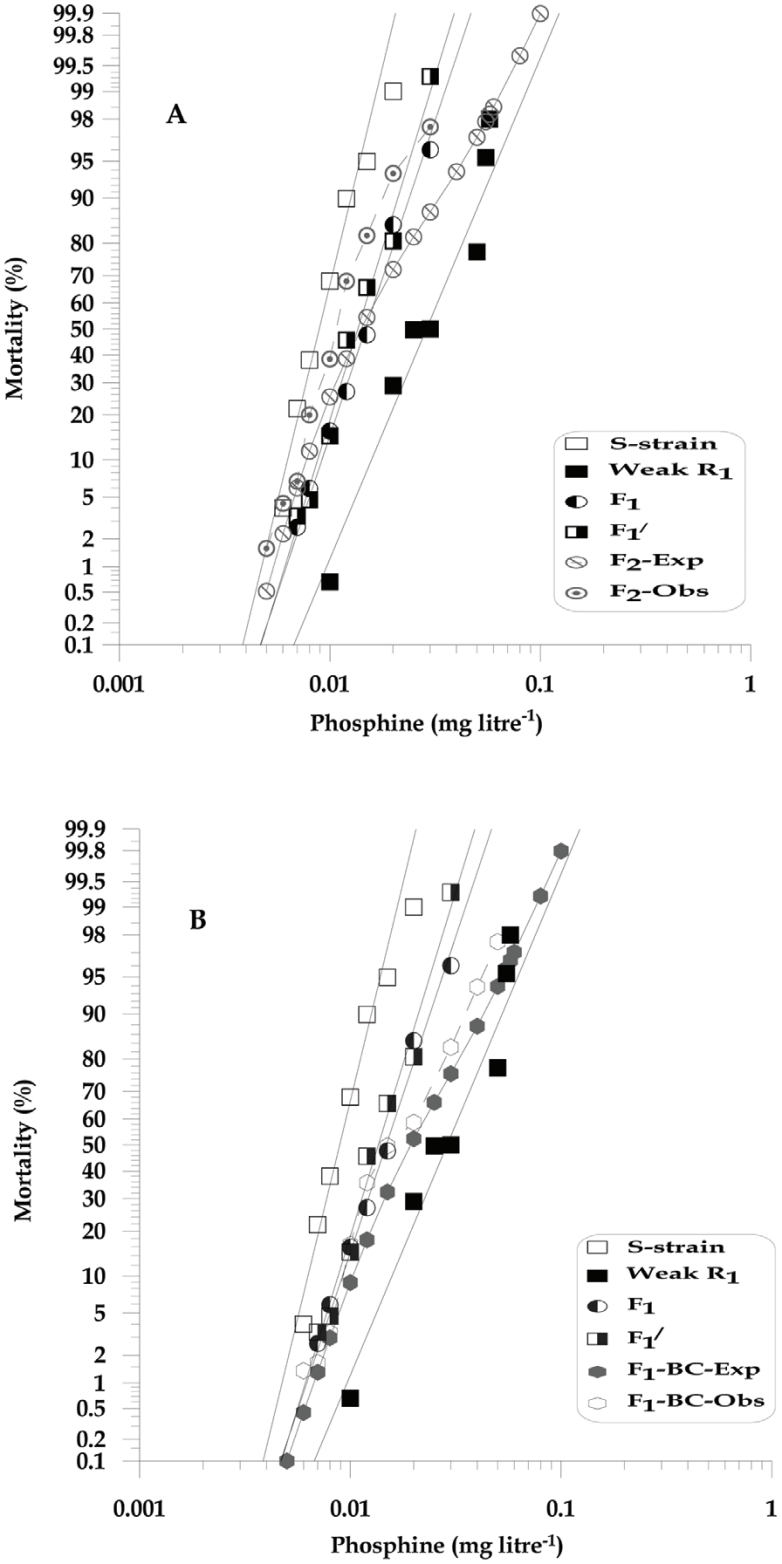
Observed responses to phosphine of *T. castaneum* adults of S-strain (QTC4) and Weak-R_1_ (QTC1012) parental strains and progeny. (A) mass F_2_ inter- strain cross and (B) F_1_-BC progeny (MIC) are shown together with expected responses calculated under the hypothesis of monogenic inheritance.

The resistance of the Weak-R_1_ (QTC1012) was 3.2× the basal tolerance of the S-strain (Table 1). The S-strain and F_1_ progeny (S-strain ♀ X Weak-R_1_ ♂) exhibited a linear probit mortality curve in response to phosphine exposure, and these responses were statistically homogenous, with a non-significant chi-square value (Table 1) indicating excellent fit to the probit model. Both the Weak-R_1_ and the F_1'_ progeny of the reciprocal cross (Weak-R_1_ ♀ X S-strain ♂) also fitted to the linear probit mortality curves (Figure 1A) as evidenced by the narrow range of fiducial limits for the LC_50_ estimates, despite the responses being statistically heterogeneous (Table 1). The modified chi-square analysis accommodated these heterogenous responses (see Materials and Methods) while testing observed and expected progeny responses for monogenic hypothesis.

**Table 1:** Probit analysis of *Tribolium castaneum* strains and their reciprocal F_1_ progenies to phosphine exposure.

Strain/Cross	*n* a	Slope ± SE	LC_50_ (95% FL) (mg litre^−1^)	LC_99.9_ (mg litre^−1^)	*χ* ^2^	df^b^	*P*	RR^c^	DD^d^
S-strain (QTC4)	803	8.518± 0.511	0.009 (0.008–0.009)	0.020	11.3	6	0.079	1	-
Weak-R_1_ (QTC1012)	1828	4.903±0.721	0.029 (0.023–0.033)	0.122	118.5	10	<0.001	3.2	-
Weak-R_2_ (QTC1389)	998	3.922±0.622	0.037 (0.027–0.048)	0.227	79.17	8	<0.001	4.1	-
Strong-R (QTC931)	1819	3.158±0.266	3.885 (3.33–4.452)	36.99	45.03	10	<0.001	431	-
F_1_ (S-strain ♀ X Weak-R_1_ ♂)	1675	6.177±0.276	0.014 (0.014–0.015)	0.047	12.62	9	0.180	1.6	−0.244
F_1'_ (Weak-R_1_ ♀ X S-strain ♂)	1648	6.732±0.450	0.014 (0.013–0.014)	0.039	18.03	9	0.0348	1.6	−0.244
F_1_ (S-strain ♀ X Strong-R ♂)	1656	7.68±1.02	0.018 (0.016–0.02)	0.045	33.75	6	<0.001	2	−0.772
F_1'_ (Strong-R ♀ X S-strain ♂)	1614	4.711±0.905	0.019 (0.013–0.025)	0.084	123	6	<0.001	2.1	−0.754
F_1_ Pooled	3270	5.554±0.700	0.018 (0.016–0.021)	0.066	212.3	14	<0.001	2	−0.772
F_1_ (Weak-R_1_ ♀ X Strong-R ♂)	1801	5.93±1.29	0.072 (0.061–0.088)	0.240	81.4	7	<0.001	2.5	−0.630
F_1'_ (Strong-R ♀ X Weak-R_1_ ♂)	1820	4.466±0.589	0.072 (0.064–0.083)	0.355	37.27	7	<0.001	2.5	−0.630
F_1_ Pooled	3621	5.013±0.594	0.073 (0.067–0.080)	0.301	119.2	20	<0.001	2.5	−0.623
F_1_ (Weak-R_1_ ♀ X Weak-R_2_ ♂)	1102	5.624±0296	0.031 (0.029–0.033)	0.110	4.72	9	0.8580	1.07	−0.452
F_1_ (Weak-R_2_ ♀ X Weak-R_1_ ♂)	1105	5.477±0.292	0.031 (0.029–0.032)	0.112	12.37	9	0.1932	1.07	−0.452
F_1_ Pooled	2207	5.550±0.208	0.031 (0.029–0.032)	0.111	17.31	20	0.6327	1.07	−0.452

a Number of insects subjected to phosphine bioassay, excluding control.

b Degrees of freedom,

c Resistance Ratio (RR) = Resistance Ratio (LC_50_ of resistant or F_1_ Hybrid/LC_50_ of susceptible/weakly resistant strain).

d Degree of Dominance (DD) = (2log LC_RS_- Log LC_R_- Log LC_S_)/(Log LC_R_-Log LC_S_).

The dose response curves of reciprocal F_1_ crosses were very close to each other and their LC_50_ values were not significantly different, as determined by the overlap of their fiducial limits (Table 1). Measuring the difference between reciprocal F_1_ responses in terms of their relative potency (the ratio of two equally effective doses) is an alternative and confirmatory approach to determine whether the responses are similar or parallel or independent [18]. The results of relative potency analysis of the reciprocal F_1_ data indicated that the F_1_ and F_1_' curves were parallel. The relative potency value was 1.08 [1.01 to 1.16, 95% fiducial limits]. A value significantly greater than 1.0 indicates that the F_1_ response data should not be combined for further statistical analysis, despite no obvious difference being observed between the two sets of data. The lack of significant maternal effects indicates that resistance to phosphine in the Weak-R_1_ is autosomally inherited.

The sensitivity of the reciprocal F_1_ populations to phosphine was nearer to the response of the S-strain than the Weak-R_1_ strain, with a degree of dominance of −0.244 (−1 = completely recessive and +1 = completely dominant). The resistance ratio of both reciprocal F_1_ progeny was 1.6-times the basal tolerance of the S-strain, suggesting that the weak resistance phenotype was expressed as an incompletely recessive trait in *T. castaneum*.

#### Number of genes conferring weak resistance

If resistance is conferred by a single recessive gene, then the resulting F_2_ progeny would consist of three possible genotypic classes (SS, SR and RR) that will give rise to two distinct phenotypes, with 75% of the progeny being sensitive and 25% resistant [22]. However, the phenotypic differences between susceptibility and resistance appear inadequate to clearly identify these phenotypes with the number of insects tested (Figure 1A). The F_2_ analysis also appears to show a consistent shift of the observed F_2_ population towards susceptibility, specifically at higher concentrations (Figure 1A) rather than the predicted response (Table S1). These deviations were significant at concentrations 0.01 to 0.02 mg l^−1^ with the maximum chi-square value of 21.4 at 0.012 mg litre^−1^(P = 4.0E-06, df = 1) and reflected in overall chi-square deviation (χ2 = 73.43, P = 2.0E-10, df = 13) (Table S1). The expectation for expression of a recessive resistance allele in the F_1_-BC progeny is that half of the F_1_-BC progeny will be heterozygous and therefore relatively susceptible, whereas the other half will be homozygous recessive and therefore resistant to phosphine. We indeed saw the expected inflection point at 50% mortality when we tested the phosphine resistance of F_1_-BC progeny (Figure 1B and Table S2) however, the results of overall goodness of fit test indicated that the observed F_1_-BC progeny response curve was significantly different (χ2 = 62.0, P = 1.0E-8, df = 13) and specifically at the concentrations 0.01 to 0.02 mg l^−1^ with the maximum individual chi-square value of 11.5 at 0.012 mg litre^−1^ (P = 0.001, df = 1) (similar to F_2_ response curve) (Table S2). Thus, while both F_2_ and F_1_-BC goodness of fit analysis reject the assumption of single gene inheritance on Weak-R_1_ strain, the visual plateau at 50% mortality level on F_1_-BC suggests some conformity to the presence of single major gene. Based on these results and the observed low level (3.2×) resistant phenotype in Weak-R_1_, we hypothesise that resistance in Weak-R_1_ may be governed by single major gene and additional minor factors.

### Inheritance of strong resistance to phosphine (MIC: S-strain X Strong-R)

#### Resistance levels, maternal effects and degree of dominance

**Figure 2:**
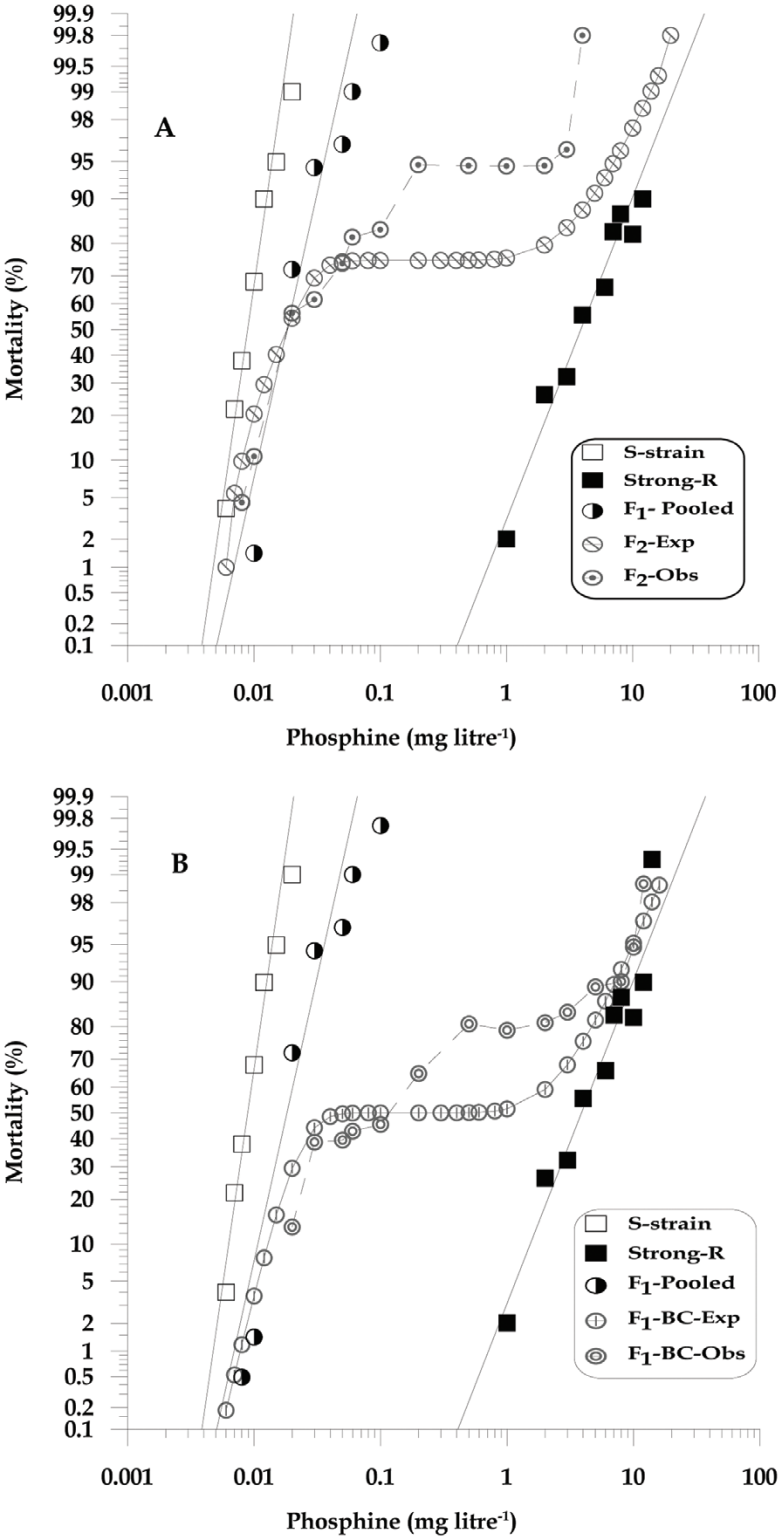
Observed responses to phosphine of *T. castaneum* adults of S-strain (QTC4) and Strong-R (QTC931) parental strains and and progeny. (A) F_2_ inter-strain cross and (B) F_1_-BC progeny (MIC) are shown together with expected responses calculated under the hypothesis of monogenic inheritance.

The resistance conferred by the Strong-R strain was 431× greater than the basal level of tolerance (Table 1). As anticipated, the response of the S-strain was homogeneous and fitted perfectly with the probit model. Although, both the Strong-R parent and the pooled reciprocal F_1_ progenies (F_1_: S-strain ♀ X Strong-R ♂ and F_1_': Strong-R ♀ X S-strain ♂) showed some degree of heterogeneous response (Table 1) the regression curves of Strong-R and its reciprocal F_1_ progeny were very close to linear (Figure 2A) with narrow fiducial limits for their LC_50_ estimates (Table 1). This indicates that the apparent heterogeneity may have resulted from the segregating genetic factors associated with other qualitative traits within the population of the strain or possibly from stochastic effects. The responses of the reciprocal F_1_ progeny were not significantly different from each other according to relative potency analysis of their LC_50_ values (Table 1). The relative potency value was 0.98 [0.95–1.23 95% fiducial limits] indicating no significant difference between the progenies. The lack of a significant difference between the F_1_ progeny of reciprocal crosses indicates that strong resistance to phosphine in *T. castaneum* is neither X-linked nor mitochondrial encoded. Because the responses of F_1_ reciprocal crosses were not distinguishable, the data were combined for subsequent statistical analyses.

The mortality responses of both reciprocal and pooled F_1_ progeny were close to that of the S-strain with a degree of dominance (DD) of −0.772 and a resistant ratio (RR) of 2.0×. The resistance factors of the F_1_ hybrids were similar (1.6× and 2.0×) regardless of whether the S-strain had been crossed with Weak-R or Strong-R, in contrast, the resistance factors in the homozygous resistant lines differed greatly, 3.2× versus 431×. (Table 1 and Figure 2A).

#### Number of genes conferring Strong resistance

If strong resistance is conferred by a single gene, a plateau at 75% mortality could be expected in the mortality curve of the F_2_ progeny whereas a plateau at 50% could be expected in the F_1_-BC curve [22]. Such a result would suggest that the same gene is responsible for both weak and strong resistance and that the difference in phenotype between the two strains is simply due to the strength of the allele present in each strain. We observed significant deviation from the single gene model. The most significant plateau occurred at about 95% mortality in the F_2_ response curve for high concentrations (0.2 to 1.0 mg litre^−1^) (Figure 2A). Lack of plateau in the observed F_2_ response curve at 75% mortality level rejected the assumption of monogenic inheritance and indicates the possibilities of multifactorial control of resistance in Strong-R strain. The overall chi-square analysis also rejected monogenic inheritance with the significant chi-square value of 23.16 (P = 0.026, df = 12) (Table S3). The observation of the individual chi-square values on observed and expected F_2_ responses at a series of concentrations showed significant differences (P<0.05) at concentrations (0.2 to 1.0 mg litre^−1^) but they disappeared, after adjustment for multiple comparisons.

If two genes contribute to resistance, the predicted phenotypic ratios would be 9∶3∶3∶1, given the simplifying assumption of complete recessivity. Thus, 9/16 (56%) would be expected to be phenotypically susceptible (i.e. genotypically either homozygous recessive or heterozygous at each of the two loci). Nineteen per cent (3/16) of each of the progeny would be homozygous resistant for one of the two loci, but not the other. The remaining 1/16 (6%) would be fully resistant as they would be homozygous recessive for both of the two loci. A precise mathematical model for a two gene system cannot be devised as we know neither the degree of resistance conferred by the hypothetical second locus nor how the two genes interact. The simplest case would be that these phenotypic classes result in plateaus on the phosphine response curve at 56%, 75% and 94% mortality. However since the genes are actually incompletely recessive a diversity of response over nine genotypic classes is to be expected and so we would expect deviation from these expectations. An inspection of Figure 2A reveals a major plateau which closely matches the prediction of 94% mortality, i.e. both genes homozygous resistant. The remaining data is insufficient to allow firm conclusions to be drawn, but it is not inconsistent with the predictions of the two gene model.

The F_1_ female progeny of reciprocal crosses between the S-strain and Strong-R were then crossed to males of the resistant parental strain, Strong-R. The resulting F_1_-BC progeny were exposed to phosphine and the mortality response was analysed to determine whether it supported a one gene or a two gene model. Analysis of the testcross progeny revealed significant deviation from the one gene model (Figure 2B), which corroborates the previous F_2_ analysis. The response curves of the single gene model and the reciprocal testcross progeny are quite distinct from each other. For instance, if the null hypothesis of monogenic inheritance is correct, then a plateau at 50% mortality is expected in the response of the F_1_-BC progeny of Strong-R. Visual examination of the observed F_1_-BC progeny response curve reveals two distinct plateaus at around 40% and 85% mortality levels, strongly suggesting the possibility of two or more major genes in governing the strong resistance phenotype in Strong-R (Figure 2B). Although we would also expect a plateau at approx 25%, the incomplete recessivity of the heterozygotes may mask the phenotypic responses at the lower doses making the plateau difficult to resolve. The results of modified chi-square analyses indicated that the mortality response curve for the F_1_-BC progeny deviated highly significantly (*P*<0.001) from the expected monogenic model (χ2 = 55.18, P = 8.057E-07, df = 14), specifically at phosphine concentrations of 0.5 and 1.0 mg litre^−1^, with the maximum χ^2^ value of 15.7 at 0.5 mg litre^−1^ (P = 7.4E-05, df = 1) (Table S4), supporting a conclusion that strong resistance is not conferred by a single major gene. Therefore, the null hypothesis of single gene inheritance can be formally rejected.

Analysis of the observed response data for the F_1_-BC and F_2_ progeny strongly supports rejection of the hypothesis of single gene inheritance in Strong-R and suggests that there are two major genes with the possibility of additional factors contributing to the strong resistance phenotype in *T. castaneum*. The inconsistency of the observed responses between the F_2_ and F_1_-BC crosses we believe is due to the number of genotypic classes expected in a two-gene model with differing degrees of response due to epistatic interactions in each case. For the F_2_, nine genotypes are expected and for the F_1_-BC only four genotypes are expected, which is perhaps why the response curves are much clearer than the F_2_.

To provide a very rough estimate of the degree of resistance provided by each genotype, we calculated the approximate LC_50_ value of the gene not shared by Weak-R_1_ and Strong-R strains from mid-point of the observed response of the F_1_-BC progeny (Figure 2B) between the plateaus and assumed a single-gene model for the Weak-R_1_ strain. In this way we calculate that the LC_50_ for this second Strong-R gene is approximately 0.2 mg litre^−1^. As the gene in the S-strain confers an LC_50_ of 0.009 mg litre^−1^ phosphine, this gives a very approximate value of ∼22× resistance factor for the Strong-R gene. When both factors are homozygous for the resistance allele, an LC_50_ of 3.9 mg litre^−1^ is evident. This suggests strongly synergistic interactions between the two resistance factors in that when homozygous separately they exhibit 3.2× and ∼22× resistance factors, but show a 431× resistance when homozygous for both factors. This rough approximation of a two gene model fits the existing data reasonably well and supports previous observations from *R. dominica* that two resistance genes work together synergistically to provide high level resistance.

### Interactions between weak and strong resistance phenotypes (MIC: Weak-R_1_ X Strong-R)

In order to understand the interactions between weak and strong phosphine resistance genes of *T. castaneum*, the Weak-R_1_ and Strong-R strains were mass crossed and their reciprocal F_1_, F_2_ and F_1_-BC progeny responses were tested. Analysis of the F_1_ hybrids also allowed complementarity and the relative dominance of alleles to be assessed.

#### Resistance levels, maternal effects and degree of dominance

**Figure 3:**
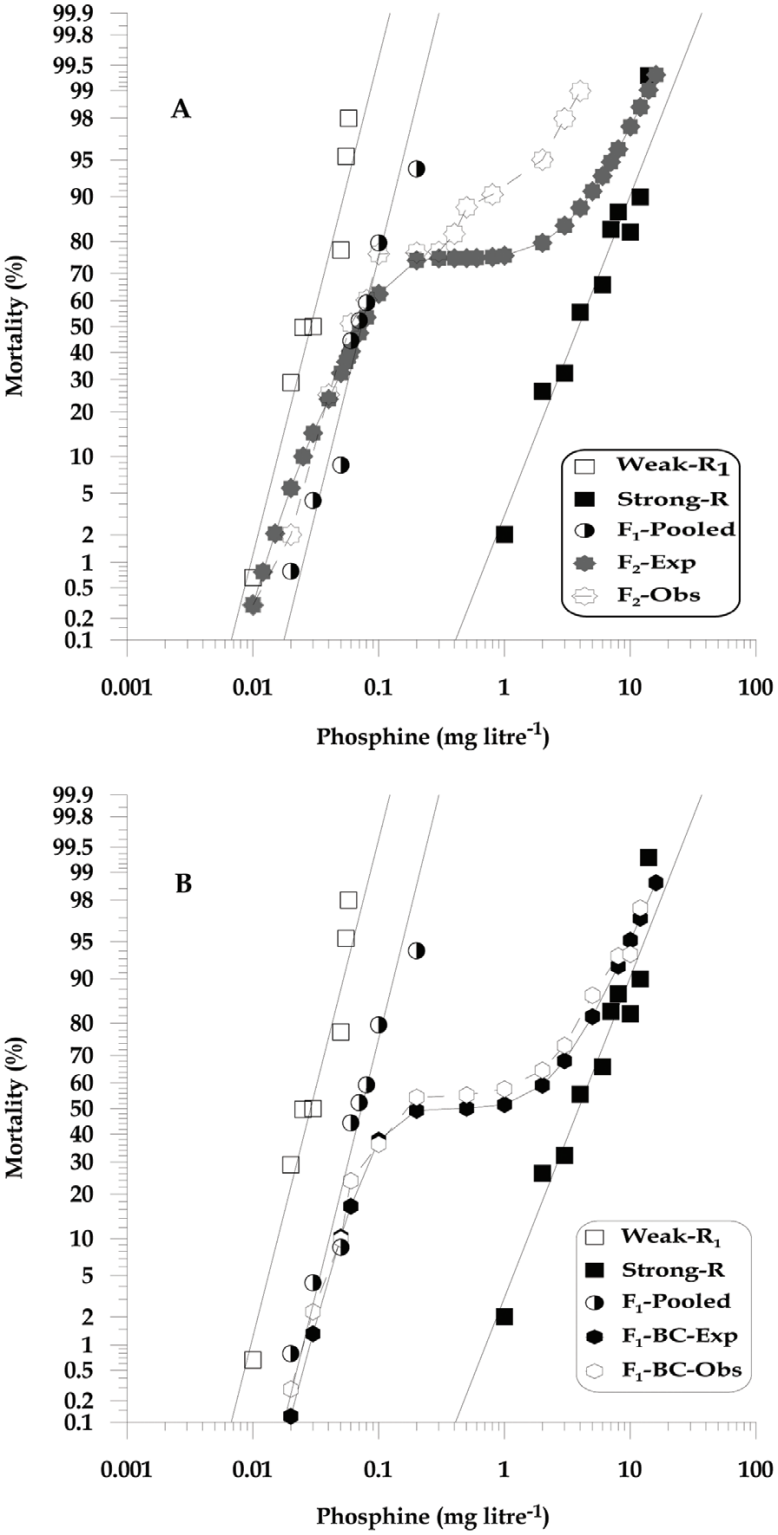
Observed responses to phosphine of *T. castaneum* adults of Weak-R_1_ (QTC1012) and Strong-R (QTC931) parental strains and progeny. (A) Mass F_2_ inter-strain cross progeny and (B) F_1_-BC progeny (MIC) are shown together with expected responses calculated under the hypothesis of monogenic inheritance.

Mortality testing revealed that the Strong-R strain had 134× higher resistances to phosphine exposure than the Weak-R_1_ strain (Table 1). Although the response to phosphine in Weak-R_1_ and Strong-R parents as well as their reciprocal F_1_ progeny (pooled) were linear with narrow fiducial range and fitted well with the probit model (Figure 3A), the statistical analysis indicated the existence of some degree of heterogenity in the parental strain (Table 1), indicating the existence of background genetic factors for other traits within the population of the stains. The dose response curves of the reciprocal F_1_ progeny (F_1_: Weak-R_1_ ♀ X Strong-R ♂) and (F_1_': Strong-R ♀ X Weak-R_1_ ♂) indicated significant overlap in their response at almost all concentrations. In addition, their respective LC_50_ and observed relative potency values were not significantly different from each other (Table 1) which confirmed that the lines were similar. The absence of maternal factors strongly indicates that the resistance phenotype is inherited autosomally and allows the response data from the F_1_ reciprocal crosses to be combined for subsequent statistical analyses. The pooled F_1_ mortality response curve lay close to that of the Weak-R_1_ with a degree of dominance (DD) of −0.623 and a resistance ratio (RR) of 2.5×, suggesting the presence of an incompletely recessive factor inherited from the Strong-R parent (Table 1 and Figure 3A).

#### Number of genes shared between weakly and strongly resistant strain

The unique resistance factor in the Strong-R strain was incompletely recessive. Therefore, a model that assumes a single gene difference between the Strong-R and Weak-R_1_ strains predicts plateaus at 75% and 50% mortality in the F_2_ and F_1_-BC response curves, respectively [22]. The observed F_2_ response curve exhibited a short plateau at around 75% mortality level at the concentrations of 0.09 to 0.3 mg litre^−1^ (Figure 3A), however, considerable divergence from theoretical expectations for single gene resistance was observed when the concentration increased to 3.0 mg litre^−1^ and it is shown in overall modified chi-square analysis (χ^2^ = 41.16, P = 8.975E-05, df = 13) (Table S5). For the individual chi-square analysis, there were no significant differences at any of the doses after Bonferroni correction, indicating conformity to single gene inheritance. Similarly, the observed F_1_-BC curve showed a significant plateau at around 50% mortality level at the concentration range of 0.2 to 0.9 mg litre^−1^ (Figure 3B) and resembled the expected curve in almost all the concentrations tested, except at 1.0 and 2.0 mg litre^−1^ indicating strong conformity to the single gene hypothesis (Figure 3B and Table S6). We accepted null hypothesis in this case by interpreting the overall shape of the response curve and it's strong indication of monogenic inheritance.

The results of F_2_ and F_1_-BC progeny analysis suggest that both Weak-R_1_ and Strong-R shared the weak resistance factor (3.2×). In addition, Strong-R appears to contain an allele at a second locus that confers 134× resistance, which is not present in Weak-R_1_. Although the null hypothesis of monogenic inheritance was rejected on the basis of significant overall chi-square values of the F_2_ (χ^2^ = 41.16, P = 8.975E-05, df = 13) and F_1_-BC, (χ^2^ = 50.95, P<4.0E-06, df = 14), the observed response of F_2_ and especially the F_1_-BC closely resembled the curves expected for single major gene inheritance between the Weak-R_1_ and Strong-R and the monogenic hypothesis is not rejected after multiple testing correction of individual comparisons. Therefore, it seems that there is a single major gene (134×) in the Strong-R that interacts synergistically with the weak resistance factor (3.2×) existing in both Weak-R_1_ and Strong-R phenotypes and contributes to high level resistance up to 431× and the small deviations observed in the F_2_ and F_1_-BC are possibly the result of the influence of the gene interactions or the existence of additional minor factors. However with the F_2_ response curve, the deviation from the expected models at the higher doses is consistent across all F_2_ inter-crosses observed regardless of parental strains (used in the present and previous study) [13], and appears consistent even in inter-cross progeny of *R. dominica* [15], [28]. The reasons for this inconsistency are not clear and possibly due to the influence of additional minor factors or modifying genes such as that described by Andres [29], lack of purity of the parental strains or experimental error due to too few of each genotype, especially those homozygous for strong resistance tested at the higher doses as in a two-gene model only 1/16th of the insects expected in the F_2_ would be homozygous strongly resistant. We also hypothesise that there could be some fitness differences in terms of development within the segregating F_2_ genotypes in a population, but the issue is yet to be resolved.

### Interactions between two weak resistance phenotypes (MIC: Weak-R_1_ X Weak-R_2_)

**Figure 4:**
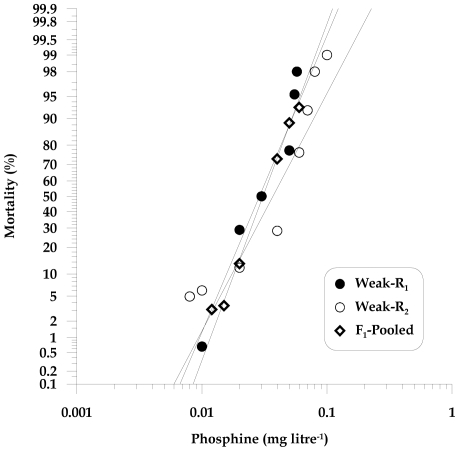
Observed responses to phosphine of *T. castaneum* adults of Weak-R_1_ (QTC1012) and Weak-R_2_ (QTC1389) parental strains and their mass inter-strain cross F_1_ progeny (MIC).

In order to confirm whether the existing weak resistance factor(s) in Weak-R_1_ are conserved in other field isolates, we crossed it with another weakly resistant strain, Weak-R_2_ and observed the response of the F_1_ progeny to phosphine. The resistance ratio of Weak-R_2_ is 4.1 whereas that for Weak-R_1_ is 3.2 (Table 1). The dose response curves of the reciprocal F_1_ progeny overlapped those of their parental strains, Weak-R_1_ and Weak-R_2_, at both low and high concentrations of phosphine (Figure 4). This indicates that both Weak-R_1_ and Weak-R_2_ contain resistance alleles of the same gene (Table 1). We also selected the most highly resistant individuals from the F_2_ population from this cross to observe whether a more strongly resistant phenotype could be selected by interbreeding homozygous weakly resistant individuals from separate field-collected strains. Weakly resistant F_2_ individuals were exposed to 0.06 mg litre^−1^ phosphine, (LC_90_ for the Weak-R strains) to ensure survival of individuals that were homozygous for the resistance factor. Surviving insects were allowed to interbreed and their resulting progeny were screened to determine whether the observed level of resistance exceeded that of the parental strains. We observed no significant increase in the level of resistance, confirming that the genetic factors responsible for weak resistance are conserved between the two strains.

### Fitness cost associated with phosphine resistance

**Figure 5:**
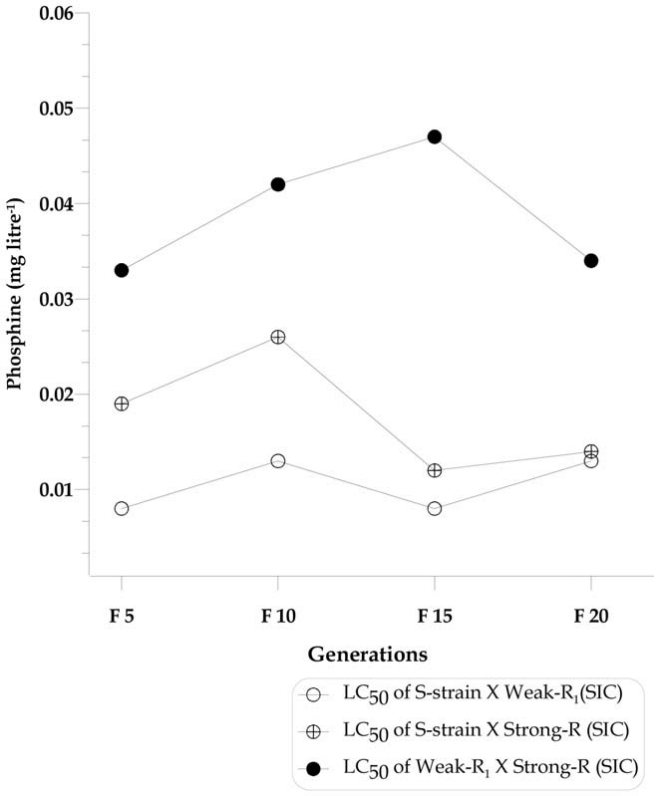
The trend of calculated LC_50_ values for the three segregating populations of *T. castaneum* adults, not exposed to phosphine, derived from the single pair inter-strain crosses (SIC) : S-strain (QTC4) X Weak-R_1_ (QTC1012), S-strain (QTC4) X Strong-R (QTC931) and S-strain (QTC4) X Strong-R (QTC931) over multiple generations.

To identify any fitness costs associated with phosphine resistance alleles, we set up several inter-strain single pair crosses: S-strain X Weak-R_1_ (SIC); S-strain X Strong-R (SIC) and Weak-R_1_ X Strong-R (SIC) and tested for their response to phosphine at F_5_, F_10_, F_15_ and F_20_ generations. The grouped non-linear regression analysis was used to compare the mortality response of different generation curves and to calculate LC values for each generation (LC_10_, LC_50_ and LC_90_). The output of this analysis clearly indicated that the dose response curves obtained in different generations (F_5_, F_10_, F_15_ & F_20_) from the three different crosses were separate (P<0.01) and not identical (Table S7 and Figures S1A, S1B, S1C), However, their mortality response to a series of phosphine concentrations followed a similar trend in certain aspects across the generations within each cross and that allowed us to use separate linear [A and C] and non-linear [B and M] parameters for each generation curve, y = A+C/(1+e ^(−B*(X-M))^) to calculate the LC values. Changes in the calculated LC_50_ values for each generation response was then evaluated for each cross (Figure 5 and Table S8) for the presence or absence of fitness cost.

No significant changes observed among LC_10_, LC_50_ and LC_90_ values over multiple generations for the cross, S-strain X Weak-R_1_ clearly indicated the absence of fitness costs for the resistance factor(s) in the Weak-R_1_ strain (Table S8, Figures 5 and S1A). However, some changes in the phenotype response observed with the other two crosses, S-strain X Strong-R and Weak-R_1_ X Strong-R over multiple generations. In the S-strain X Strong-R cross, the LC_50_ and LC_90_ values for the F_5_ were 0.019 and 0.07 mg litre^−1^, respectively, which decreased to 0.012 and 0.034 mg litre^−1^ at F_15_, and then to 0.014 and 0.047 mg litre^−1^, respectively, at F_20_, whilst the LC_10_ values remained unchanged (Table S8, Figures 5 and S1B), but this trend was not monotonic as the LC_10_ and LC_50_ for the F_10_ were higher than the F_5_ and so firm conclusions for the existence of a fitness cost are difficult to make on the basis of the phenotype data presented. In the Weak-R_1_ X Strong-R cross, while there were changes at each generation, the overall trend showed no change. The calculated LC_10_, LC_50_ or LC_90_ values of the F_5_, F_10_ and F_20_ generations remained in the ranges of 0.017–0.023 mg litre^−1^, 0.033–0.042 mg litre^−1^ and 0.083–0.98 mg litre^−1^, respectively, suggesting the absence of any observable of fitness cost associated with the resistance factor observed in the Strong-R strain responsible for high-level resistance when in the weakly resistant background (Table S8, Figures 5 and S1C).

## Discussion

Our hypothesis at the beginning of this research was that inheritance of resistance to phosphine in *T. castaneum* should resemble the inheritance of phosphine resistance in *R. dominica*, i.e. that the Weak-R phenotype is predominantly controlled by a single major gene, *rph1*. Furthermore, *rph1* plus a second factor, *rph2*, account for most of the resistance of the Strong-R phenotype [15], [28], [30]. Our results indicated that the situation in *T. castaneum* does indeed resemble that of *R. dominica*. Evidence from the inheritance and complementation analyses of the two Weak-R strains in this study revealed that low-level resistance to phosphine in *T. castaneum* is most likely governed by a single major gene, although one or more minor factors appear to contribute to resistance as well. The analysis of the S-strain X Strong-R cross indicated that strong resistance to phosphine in *T. castaneum* is conferred by two major genes, again with some influence from additional factors. Our results are consistent with those of Ansell [12], Bekon *et al.*, [11] and Bengston *et al.*, [13] who concluded that weak resistance was controlled by one major gene in *T. castaneum* but other factors may also be present. Ansell [12] also concluded that high level resistance to phosphine in *T. castaneum* from Pakistan appeared to be mediated by two major genes. Limited studies in two other insect species, *R. dominica* and *Sitophilus oryzae* (Linneaus), also suggest that strong resistance to phosphine is predominantly governed by two major genes [3], [15], [28], [31].

The test crosses (F_1_-BC) between Weak-R_1_ and Strong-R revealed that both phenotypes share the low level resistance factor conferring a weak effect of about 3.2×, while the Strong-R has an additional major gene that confers a higher resistance of about 134×, which is not present in Weak-R_1_. The very high level of resistance (431×) shown by the Strong-R phenotype appears to be a result of the epistatic synergism of these two genes. Synergism of two major genes producing the Strong-R phenotype, one of which is allelic with the weak resistance gene, has also been observed in *R. dominica* [12], [15], [30]. The similarity between the *T. castaneum* genotypes and that of *R. dominica*, insects from quite distinct families of Coleoptera, suggests that the mechanisms involved in phosphine resistance are associated with fundamental biochemical processes.

The cross between the two weak resistance strains, Weak-R_1_ and Weak-R_2_ (Figure 4), demonstrated that low level resistance in *T. castaneum* is well conserved, i.e. that the resistance in these strains is allelic. Evidence from national resistance surveys [14] indicates that the resistance expressed by our test strains, Weak-R_1_ and Weak-R_2_, is typical of field resistance in Australia.

The level of resistance observed in both Weak-R strains is in the range previously reported for *T. castaneum* using similar bioassay methods [13], [32]. However, the level of resistance shown by the Strong-R (431×) is higher than previously reported for this species, 186.2× at LD_50_ from Brazil and 125× at LD_99_ from India [4], [7], but comparable to levels reported in other grain insect pest species including *R. dominica* [15], [33], *Oryzaephilus surinamensis* (Linneaus) [34], and *Cryptolestes ferrugineus* (Stephens) [9], [35].

Resistance in both the Weak-R and Strong-R strains was inherited autosomally and there was no evidence for maternal effects. These results eliminate the possibility that resistant insects have mutations in the mitochondrial genome, despite mitochondria being proposed as the primary target of phosphine action [36]. Autosomal inheritance of phosphine resistance was also observed in previous studies of phosphine resistance in *T. castaneum* [12], [13], *R. dominica* and *S. oryzae* [15], [31]. Both weak and strong resistance genes are expressed as an incompletely recessive trait and therefore, the resulting heterozygotes are more tolerant than the sensitive phenotype but can easily be killed with higher concentration and exposure time. This can potentially slow down the rate of selection of resistance alleles in the field compared to resistance genes expressed as a dominant trait.

A potentially important characteristic of resistance genes is the possible association of resistance with reduced fitness. Our study indicated the possible association of a weak fitness deficit associated with the Strong-R gene when crossed into a susceptible background (i.e. into the S-strain) but not when crossed into a Weak-R background. However, these results were not seen as a monotonic trend, making it difficult to base firm conclusions on the existence a fitness cost, even though the observed LC_50_ values fluctuated over more than 50% between F_10_ and F_15_ generations of the S-strain x Strong-R cross. Therefore it appears that there is no clearly observable fitness deficit associated with weak and strong resistance to phosphine in *T. castaneum*. Previous genetic analyses provided no indication of any fitness deficit associated with resistance to phosphine in *R. dominica* [14], [30] or *T. castaneum* [37]. In a different approach to measuring fitness, Pimentel *et al.* [7] and Sousa *et al.* [38] measured demographic parameters of field strains of several major grain insect pests and found that a fitness deficit appeared to be associated with resistance. However, we could not make firm conclusions on fitness from this study, as our statistical methods lack resolving power when only using phenotypic data to infer changes in genotype frequencies in a segregating population. To address this problem, identification of DNA markers tightly linked to Weak-R and Strong-R genes is currently in progress and our next step will be to use these markers to support our phenotypic fitness data.

### Conclusion

Insecticide resistance is an evolutionary response to selection. Effective resistance management relies on an understanding of this process. The rate of resistance development is determined by interacting abiotic and biotic factors. There were three key findings from this study. First, phosphine resistance in *T. castaneum* is controlled by at least two major autosomal genes that are almost incompletely recessive in expression. Second, resistance is fully expressed when both genes are homozygous producing a synergistic effect that results in the Strong-R phenotype. Third, it appears that while there is change in the phenotypic response over multiple generations in segregating cross, it is not consistent, making firm conclusions for the existence of a fitness cost is difficult.

The practical importance of recessive expression of resistance genes is that heterozygote adults can be controlled with only a small increase in phosphine concentration or by extending the fumigation period. Further research is needed to determine whether the expression of resistance genes is incompletely recessive in other life stages. Despite the significant advantage conferred by the Strong-R phenotype during exposure to phosphine, this resistance is still uncommon in *T. castaneum* populations in Australia (Collins PJ, unpublished). Multi-gene resistance has also been associated with a slow rate of development in other species/insecticide systems [39]. Furthermore, the response to selection of multiple resistance genes in terms of gene frequencies in the resistant population depends on multiple genetic factors such as dominance, gene interactions and relative fitness [40], [41], making it very difficult to predict the outcome of various resistance management tactics. The observed fitness results of this study do not appear to result in a major change in the level of susceptibility in weak or strong resistance to phosphine over multiple generations without selection, thus any fitness cost associated with phosphine resistance is either too minor to resolve or does not exist. This suggests that mitigation strategies such as temporal rotation of chemicals [42], stable zone strategy [43] and use of refuges may not be as effective for reducing the phosphine resistant population since these tactics rely mainly on a significant fitness cost associated with resistance.

The genotypes and phenotypes identified in this research will be used to identify the genomic locations of the major genes conferring strong and weak resistance in *T. castaneum*. Molecular markers for each of the genetic loci will be able to give us a much greater resolving power for gene interactions, fitness analyses and provide insight into the underlying mechanisms of resistance.

## Supporting Information

Figure S1 **The observed fitness response curves of three segregating population obtained from single pair inter-strain crosses, S-strain X Weak-R_1_(A), S-strain X Strong-R (B), and Weak-R_1_ X Strong-R (C) at discrete generations F_5_, F_10_, F_15_ and F_20_.** The curve obtained by fitting the per cent mortality values of observed response of each population at graded series of phosphine concentrations against non-linear “S” shaped regression curve. The parameters (linear and non-linear) from the curve equation y = A+C/(1+e ^(−B*(X-M))^) was used to calculate the lethal concentrations (LC).
(TIF)

Table S1 **Chi-square analysis for testing single gene model inheritance of F_2_ progeny obtained from the mass inter-strain cross (MIC) of the parental strains, S-strain and Weak-R_1_ with their observed mortality response.**
(DOCX)

Table S2 **Chi-square analysis for testing single gene model inheritance of F_1_-BC progeny obtained from the mass inter-strain cross (MIC) of the parental strains, S-strain and Weak-R_1_ with their observed mortality response.**
(DOCX)

Table S3 **Chi-square analysis for testing single gene model inheritance of F_2_ progeny obtained from the mass inter-strain cross (MIC) of the parental strains, S-strain and Strong-R with their observed mortality response.**
(DOCX)

Table S4 **Chi-square analysis for testing single gene model inheritance of F_1_-BC progeny obtained from the mass inter-strain cross (MIC) of the parental strains, S-strain and Strong-R with their observed mortality response.**
(DOCX)

Table S5 **Chi-square analysis for testing single gene model inheritance of F_2_ progeny obtained from the mass inter-strain cross (MIC) of the parental strains, Weak-R_1_ and Strong-R with their observed mortality response.**
(DOCX)

Table S6 **Chi-square analysis for testing single gene model inheritance of F_1_-BC progeny obtained from the mass inter-strain cross (MIC) of the parental strains, Weak-R_1_ and Strong-R with their observed mortality response.**
(DOCX)

Table S7 **Summary of non-linear regression analysis performed on phosphine unexposed** ***Tribolium castaneum*** **population, obtained from single pair inter-strain crosses (SIC); S-strain X Weak-R_1_, S-strain X Strong-R, and Weak-R_1_ X Strong-R segregating for weak, strong and the both weak and strong resistant alleles, respectively over a period of twenty generations.**
(DOCX)

Table S8 **The changes in the calculated LC_10_, LC_50_ and LC_90_ values from discrete generations F_5_, F_10_, F_15_ and F_2_ of the phosphine unexposed** ***Tribolium castaneum*** **population, obtained from single pair inter-strain crosses (SIC); S-strain X Weak-R_1_, S-strain X Strong-R, and Weak-R_1_ X Strong-R segregating for weak, strong and the both weak and strong resistant alleles, respectively.**
(DOCX)
