# Supplementary figures and images for: Phosphine Resistance in the Rust Red Flour Beetle, Tribolium castaneum (Coleoptera: Tenebrionidae): Inheritance, Gene Interactions and Fitness Costs
Source: PLoS One. 2012 Feb 21;7(2):e31582. doi: 10.1371/journal.pone.0031582 (PMC3283673; doi:10.1371/journal.pone.0031582)

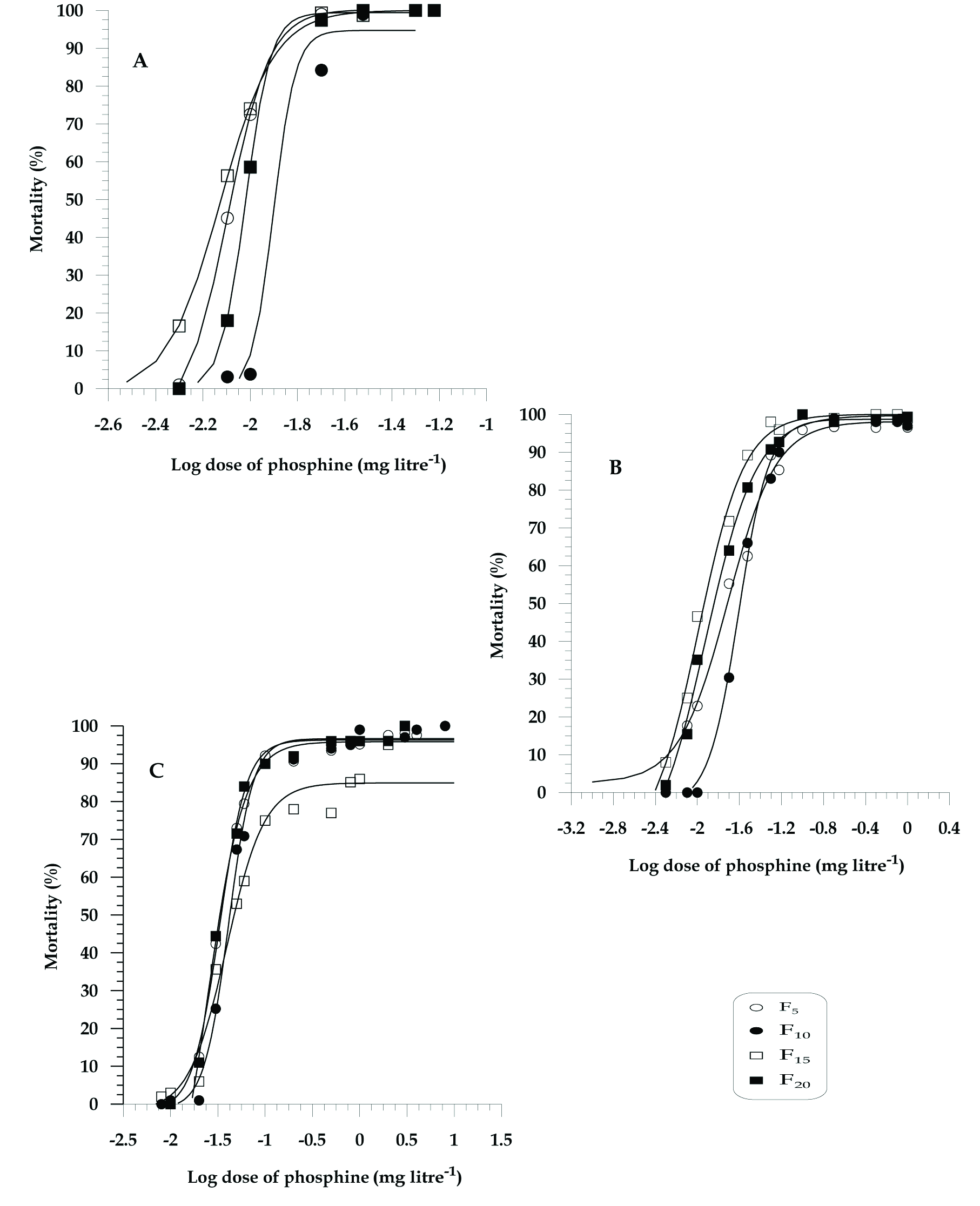

Supplement: Figure S1 — The observed fitness response curves of three segregating population obtained from single pair inter-strain crosses, S-strain X Weak-R1(A), S-strain X Strong-R (B), and Weak-R1 X Strong-R (C) at discrete generations F5, F10, F15 and F20. The curve obtained by fitting the per cent mortality values of observed response of each population at graded series of phosphine concentrations against non-linear “S” shaped regression curve. The parameters (linear and non-linear) from the curve equation y = A+C/(1+e (−B*(X-M))) was used to calculate the lethal concentrations (LC). (TIF) [file pone.0031582.s001.tif]
