# Supplementary material for: Phosphine Resistance in the Rust Red Flour Beetle, Tribolium castaneum (Coleoptera: Tenebrionidae): Inheritance, Gene Interactions and Fitness Costs
Source: PLoS One. 2012 Feb 21;7(2):e31582. doi: 10.1371/journal.pone.0031582 (PMC3283673; doi:10.1371/journal.pone.0031582)
Supplement: Table S1 — Chi-square analysis for testing single gene model inheritance of F2 progeny obtained from the mass inter-strain cross (MIC) of the parental strains, S-strain and Weak-R1 with their observed mortality response. (DOCX) [file pone.0031582.s002.docx]

**Table S1**. Chi-square analysis for testing single gene model inheritance of F_2_ progeny from mass inter-strain cross of the parental strains, QTC4 (S-strain) and QTC1012 (Weak-R_1_) with their observed mortality response.

| **Dose  (mg litre^-1^)** | **No. tested** | **Mortality Observed** | **Chi-square analysis** | | |
| --- | --- | --- | --- | --- | --- |
|  |  |  | **Mortality**  **Expected** | **Modified  *χ ^2^*** | ***P* value** |
| 0.005 | 252 | 4 | 1.3 | 1.3 | 0.249 |
| 0.006 | 251 | 11 | 5.7 | 1.2 | 0.273 |
| 0.007 | 250 | 17 | 15.0 | 0.1 | 0.793 |
| 0.008 | 250 | 50 | 29.0 | 4.0 | 0.045 |
| 0.01 | 250 | 96 | 63.3 | 5.3 | 0.022 |
| 0.012 | 250 | 170 | 96.3 | 21.4*** | 4.0E-06 |
| 0.015 | 250 | 205 | 136.3 | 17.8*** | 3.0E-05 |
| 0.02 | 250 | 234 | 179.8 | 13.5** | 2.3E-04 |
| 0.03 | 250 | 244 | 218.5 | 5.5 | 0.019 |
| 0.05 | 250 | 250 | 242.4 | 1.8 | 0.177 |
| 0.06 | 250 | 250 | 246.3 | 0.9 | 0.348 |
| 0.07 | 251 | 251 | 249.1 | 0.4 | 0.509 |
| 0.08 | 248 | 248 | 247.1 | 0.2 | 0.642 |
|  |  |  | Overall ***χ ^2^*** | 73.43*** | 2.0E-10 (13 df) |

* Significant (*P* < 0.05); ** Significant (*P* < 0.01); *** Significant (*P* < 0.001) after Bonferroni adjustment for multiple comparisons.
