# Supplementary material for: Phosphine Resistance in the Rust Red Flour Beetle, Tribolium castaneum (Coleoptera: Tenebrionidae): Inheritance, Gene Interactions and Fitness Costs
Source: PLoS One. 2012 Feb 21;7(2):e31582. doi: 10.1371/journal.pone.0031582 (PMC3283673; doi:10.1371/journal.pone.0031582)
Supplement: Table S2 — Chi-square analysis for testing single gene model inheritance of F1-BC progeny obtained from the mass inter-strain cross (MIC) of the parental strains, S-strain and Weak-R1 with their observed mortality response. (DOCX) [file pone.0031582.s003.docx]

**Table S2.** Chi-square analysis for testing single gene model inheritance of F_1_-BC progeny from mass inter-strain cross of the parental strains, QTC4 (S-strain) and QTC1012 (Weak-R_1_) with their observed mortality response.

| **Dose  (mg litre^-1^)** | **No. tested** | **Mortality Observed** | **Chi-square analysis** | | |
| --- | --- | --- | --- | --- | --- |
|  |  |  | **Mortality**  **Expected** | **Modified  *χ ^2^*** | ***P* value** |
| 0.005 | 296 | 3 | 0.3 | 3.5 | 0.062 |
| 0.006 | 293 | 10 | 1.3 | 8.2* | 0.004 |
| 0.007 | 296 | 11 | 3.9 | 1.9 | 0.173 |
| 0.008 | 297 | 16 | 8.8 | 0.9 | 0.354 |
| 0.01 | 296 | 55 | 26.4 | 4.8 | 0.028 |
| 0.012 | 295 | 111 | 52.1 | 11.5** | 0.0007 |
| 0.015 | 294 | 152 | 94.7 | 7.2 | 0.007 |
| 0.02 | 296 | 180 | 155.1 | 1.2 | 0.275 |
| 0.03 | 295 | 251 | 195.1 | 6.7 | 0.010 |
| 0.04 | 294 | 282 | 222.6 | 9.3* | 0.002 |
| 0.05 | 298 | 297 | 261.4 | 5.6 | 0.018 |
| 0.06 | 293 | 292 | 282.6 | 1.3 | 0.264 |
|  |  |  | Overall ***χ ^2^*** | 62.0*** | 1.0E-08(12 df) |

* Significant (*P* < 0.05); ** Significant (*P* < 0.01); *** Significant (*P* < 0.001) after Bonferroni adjustment for multiple comparisons.
