# Supplementary material for: Phosphine Resistance in the Rust Red Flour Beetle, Tribolium castaneum (Coleoptera: Tenebrionidae): Inheritance, Gene Interactions and Fitness Costs
Source: PLoS One. 2012 Feb 21;7(2):e31582. doi: 10.1371/journal.pone.0031582 (PMC3283673; doi:10.1371/journal.pone.0031582)
Supplement: Table S3 — Chi-square analysis for testing single gene model inheritance of F2 progeny obtained from the mass inter-strain cross (MIC) of the parental strains, S-strain and Strong-R with their observed mortality response. (DOCX) [file pone.0031582.s004.docx]

**Table S3**. Chi-square analysis for testing single gene model inheritance of F_2_ progeny from mass inter-strain cross of parental strains, QTC4 (S-strain) and QTC931 (Strong-R) with observed mortality.

| **Dose  (mg litre^-1^)** | **No. tested** | **Mortality Observed** | **Chi-square analysis** | | |
| --- | --- | --- | --- | --- | --- |
|  |  |  | **Mortality**  **Expected** | **Modified  *χ ^2^*** | ***P* value** |
| 0.008 | 199 | 9 | 19.6 | 0.7 | 0.399 |
| 0.01 | 196 | 21 | 40.0 | 1.3 | 0.262 |
| 0.02 | 197 | 111 | 107.2 | 0.0 | 0.854 |
| 0.03 | 203 | 125 | 140.6 | 0.6 | 0.428 |
| 0.05 | 201 | 149 | 150.0 | 0.0 | 0.957 |
| 0.06 | 202 | 165 | 151.3 | 0.6 | 0.458 |
| 0.1 | 201 | 168 | 150.7 | 0.9 | 0.348 |
| 0.2 | 205 | 194 | 153.8 | 4.7 | 0.030 |
| 0.5 | 202 | 191 | 151.6 | 4.6 | 0.033 |
| 1.0 | 200 | 189 | 151.6 | 4.3 | 0.039 |
| 2.0 | 202 | 191 | 160.7 | 3.1 | 0.077 |
| 3.0 | 204 | 196 | 171.4 | 2.5 | 0.117 |
|  |  |  | Overall ***χ ^2^*** | 23.16* | 0.026 (12 df) |

* Significant (*P* < 0.05); ** Significant (*P* < 0.01); *** Significant (*P* < 0.001) after Bonferroni adjustment for multiple comparisons.
