# Supplementary material for: Phosphine Resistance in the Rust Red Flour Beetle, Tribolium castaneum (Coleoptera: Tenebrionidae): Inheritance, Gene Interactions and Fitness Costs
Source: PLoS One. 2012 Feb 21;7(2):e31582. doi: 10.1371/journal.pone.0031582 (PMC3283673; doi:10.1371/journal.pone.0031582)
Supplement: Table S4 — Chi-square analysis for testing single gene model inheritance of F1-BC progeny obtained from the mass inter-strain cross (MIC) of the parental strains, S-strain and Strong-R with their observed mortality response. (DOCX) [file pone.0031582.s005.docx]

**Table S4**. Chi-square analysis for testing single gene model inheritance of F_1_-BC progeny from mass inter-strain cross of parental strains, QTC4 (S-strain) and QTC931 (Strong-R) with observed mortality.

| **Dose  (mg litre^-1^)** | **No. tested** | **Mortality Observed** | **Chi-square analysis** | | |
| --- | --- | --- | --- | --- | --- |
|  |  |  | **Mortality**  **Expected** | **Modified  *χ ^2^*** | ***P* value** |
| 0.02 | 1178 | 51 | 132.8 | 5.8 | 0.124 |
| 0.03 | 432 | 167 | 191.2 | 0.6 | 0.453 |
| 0.05 | 406 | 160 | 201.5 | 1.7 | 0.189 |
| 0.06 | 416 | 178 | 207.6 | 0.9 | 0.355 |
| 0.1 | 403 | 183 | 201.5 | 0.3 | 0.557 |
| 0.2 | 404 | 262 | 202.0 | 3.6 | 0.057 |
| 0.5 | 417 | 336 | 209.0 | 15.7 ** | 7.4E-07 |
| 1.0 | 402 | 317 | 207.3 | 12.2 ** | 0.0005 |
| 2.0 | 393 | 318 | 232.1 | 7.9 | 0.005 |
| 3.0 | 413 | 345 | 281.2 | 4.6 | 0.032 |
| 5.0 | 395 | 351 | 323.0 | 1.4 | 0.245 |
| 8.0 | 402 | 361 | 369.7 | 0.3 | 0.613 |
| 10.0 | 400 | 378 | 380.5 | 0.0 | 0.852 |
| 12.0 | 397 | 391 | 384.9 | 0.3 | 0.569 |
|  |  |  | Overall ***χ ^2^*** | 55.18*** | 8.05E-07(14 df) |

* Significant (*P* < 0.05); ** Significant (*P* < 0.01); *** Significant (*P* < 0.001) after Bonferroni adjustment for multiple comparisons.
