# Supplementary material for: Phosphine Resistance in the Rust Red Flour Beetle, Tribolium castaneum (Coleoptera: Tenebrionidae): Inheritance, Gene Interactions and Fitness Costs
Source: PLoS One. 2012 Feb 21;7(2):e31582. doi: 10.1371/journal.pone.0031582 (PMC3283673; doi:10.1371/journal.pone.0031582)
Supplement: Table S5 — Chi-square analysis for testing single gene model inheritance of F2 progeny obtained from the mass inter-strain cross (MIC) of the parental strains, Weak-R1 and Strong-R with their observed mortality response. (DOCX) [file pone.0031582.s006.docx]

**Table S5**. Chi-square analysis for testing single gene model inheritance of F_2_ progeny from mass inter-strain cross of parental strains, QTC1012 (Weak-R_1_) and QTC931 (Strong-R) with their observed mortality.

| **Dose  (mg litre^-1^)** | **No. tested** | **Mortality Observed** | **Chi-square analysis** | | |
| --- | --- | --- | --- | --- | --- |
|  |  |  | **Mortality**  **Expected** | **Modified  *χ ^2^*** | ***P* value** |
| 0.02 | 1208 | 15 | 17.9 | 0.06 | 0.999 |
| 0.04 | 305 | 78 | 72.3 | 0.1 | 0.772 |
| 0.06 | 305 | 157 | 122.9 | 2.2 | 0.135 |
| 0.08 | 305 | 185 | 163.6 | 0.9 | 0.355 |
| 0.1 | 314 | 240 | 196.6 | 3.6 | 0.057 |
| 0.2 | 298 | 229 | 221.4 | 0.1 | 0.705 |
| 0.5 | 303 | 267 | 227.4 | 3.9 | 0.048 |
| 0.8 | 303 | 274 | 228.4 | 5.2 | 0.022 |
| 1.0 | 300 | 264 | 227.4 | 3.4 | 0.063 |
| 2.0 | 302 | 287 | 240.2 | 6.3 | 0.012 |
| 3.0 | 300 | 294 | 252.1 | 6.2 | 0.013 |
| 4.0 | 299 | 296 | 262.8 | 4.9 | 0.027 |
| 5.0 | 300 | 300 | 272.7 | 4.2 | 0.039 |
|  |  |  | Overall ***χ ^2^*** | 41.16*** | 8.97E-05 (13 df) |

* Significant (*P* < 0.05); ** Significant (*P* < 0.01); *** Significant (*P* < 0.001) after Bonferroni adjustment for multiple comparisons.
