# Supplementary material for: Phosphine Resistance in the Rust Red Flour Beetle, Tribolium castaneum (Coleoptera: Tenebrionidae): Inheritance, Gene Interactions and Fitness Costs
Source: PLoS One. 2012 Feb 21;7(2):e31582. doi: 10.1371/journal.pone.0031582 (PMC3283673; doi:10.1371/journal.pone.0031582)
Supplement: Table S6 — Chi-square analysis for testing single gene model inheritance of F1-BC progeny obtained from the mass inter-strain cross (MIC) of the parental strains, Weak-R1 and Strong-R with their observed mortality response. (DOCX) [file pone.0031582.s007.docx]

**Table S6**. Chi-square analysis for testing single gene model inheritance of F_1_-BC progeny from mass inter-strain cross of parental strains, QTC1012 (Weak-R_1_) and QTC931 (Strong-R) with their observed mortality.

| **Dose  (mg litre^-1^)** | **No. tested** | **Mortality Observed** | **Chi-square analysis** | | |
| --- | --- | --- | --- | --- | --- |
|  |  |  | **Mortality**  **Expected** | **Modified  *χ ^2^*** | ***P* value** |
| 0.04 | 2811 | 22 | 34.7 | 0.9 | 0.925 |
| 0.06 | 699 | 71 | 117.3 | 4.2 | 0.040 |
| 0.08 | 699 | 168 | 202.8 | 1.6 | 0.205 |
| 0.1 | 702 | 256 | 264.8 | 0.1 | 0.766 |
| 0.2 | 702 | 383 | 346.1 | 1.5 | 0.223 |
| 0.5 | 702 | 389 | 351.9 | 1.5 | 0.220 |
| 0.8 | 699 | 404 | 354.8 | 2.7 | 0.103 |
| 1.0 | 700 | 454 | 361.0 | 9.5* | 0.002 |
| 2.0 | 706 | 518 | 417.0 | 11.4* | 0.0007 |
| 4.0 | 705 | 612 | 534.4 | 8.9 | 0.003 |
| 6.0 | 499 | 466 | 430.3 | 4.1 | 0.042 |
| 8.0 | 501 | 469 | 460.7 | 0.4 | 0.551 |
| 10.0 | 500 | 488 | 475.7 | 1.3 | 0.262 |
| 12.0 | 500 | 500 | 484.8 | 3.0 | 0.083 |
|  |  |  | Overall ***χ ^2^*** | 50.95*** | 4.0E-06 (14 df) |

* Significant (*P* < 0.05); ** Significant (*P* < 0.01); *** Significant (*P* < 0.001) after Bonferroni adjustment for multiple comparisons.
