# Supplementary material for: Phosphine Resistance in the Rust Red Flour Beetle, Tribolium castaneum (Coleoptera: Tenebrionidae): Inheritance, Gene Interactions and Fitness Costs
Source: PLoS One. 2012 Feb 21;7(2):e31582. doi: 10.1371/journal.pone.0031582 (PMC3283673; doi:10.1371/journal.pone.0031582)
Supplement: Table S7 — Summary of non-linear regression analysis performed on phosphine unexposed Tribolium castaneum population, obtained from single pair inter-strain crosses (SIC); S-strain X Weak-R1, S-strain X Strong-R, and Weak-R1 X Strong-R segregating for weak, strong and the both weak and strong resistant alleles, respectively over a period of twenty generations. (DOCX) [file pone.0031582.s008.docx]

**Table S7.** Summary of non-linear regression analysis of the dose-mortality response values obtained from three single pair inter strain crosses over multiple generations in *Tribolium castaneum.*

| **Cross** | **Generation** | **Separate Nonlinear Parameter estimates *±* SE**  Curve model: y= A+C/(1+e ^(-B*(X-M))^) | | | | **RSS** | **df** | **R^2^** | ***P*** |
| --- | --- | --- | --- | --- | --- | --- | --- | --- | --- |
|  |  | **B** | **M** | **C** | **A** |  |  |  |  |
| S-strain X Weak-R_1_ (SIC) | F_5_ | 11.36 ± 3.62 | -2.09 ± *­*0.029 | 108.7 ± 13.4 | -8.5 ± 11.9 |  |  |  |  |
|  | F_10_ | 21.6 ± 15.0 | -1.90 ± 0.069 | 96.13 ± 5.16 | -1.44 ± 3.63 | 178.8 | 9 | 1.00 | <0.001 |
|  | F_15_ | 8.78 ± 3.87 | -2.13 ± 0.074 | 101.3 ± 26.4 | -1.3 ± 25.2 |  |  |  |  |
|  | F_20_ | 18.99 ± 4.20 | -2.02 ± 0.011 | 99.93 ± 5.34 | -0.52 ± 4.80 |  |  |  |  |
|  | F_5_ | 4.32 ± 0.584 | -1.71± 0.056 | 95.68 ± 8.74 | 2.40 ± 8.39 |  |  |  |  |
| S-strain X Strong-R (SIC) | F_10_ | 6.92 ± 0.590 | -1.61 ± 0.014 | 102.1 ± 2.30 | -3.39 ± 2.04 | 297.1 | 45 | 1.00 | <0.001 |
|  | F_15_ | 4.55 ± 0.491 | -1.99 ± 0.047 | 115.1 ± 10.2 | -15.13 ± 9.96 |  |  |  |  |
|  | F_20_ | 4.09 ± 0.400 | -1.92 ± 0.047 | 119.4 ± 9.32 | -19.77 ± 9.02 |  |  |  |  |
|  | F_5_ | 5.23 ± 1.28 | -1.60 ± 0.144 | 133.4 ± 46.8 | -37.6 ± 46.3 |  |  |  |  |
| Weak-R_1_ X Strong-R (SIC) | F_10_ | 7.71 ± 0.86 | -1.39± 0.018 | 97.98 ± 2.63 | -1.36 ± 2.25 | 587.8 | 41 | 1.00 | 0.002 |
|  | F_15_ | 4.99 ± 0.674 | -1.41 ± 0.029 | 86.97 ± 3.76 | -2.02 ± 3.00 |  |  |  |  |
|  | F_20_ | 6.98 ± 0.777 | -1.48 ± 0.019 | 98.25 ± 2.98 | -1.98 ± 2.51 |  |  |  |  |

*All the separate non linear parameters estimates were significant at *P*<0.01 by Fisher’s test

SIC = Single Pair Inter strain cross

RSS = Residual Sum of Square; df = Degree of freedom; R^2^ = Total sum of Square/Regression sum of Square
