# Supplementary material for: Phosphine Resistance in the Rust Red Flour Beetle, Tribolium castaneum (Coleoptera: Tenebrionidae): Inheritance, Gene Interactions and Fitness Costs
Source: PLoS One. 2012 Feb 21;7(2):e31582. doi: 10.1371/journal.pone.0031582 (PMC3283673; doi:10.1371/journal.pone.0031582)
Supplement: Table S8 — The changes in the calculated LC10, LC50 and LC90 values from discrete generations F5, F10, F15 and F2 of the phosphine unexposed Tribolium castaneum population, obtained from single pair inter-strain crosses (SIC); S-strain X Weak-R1, S-strain X Strong-R, and Weak-R1 X Strong-R segregating for weak, strong and the both weak and strong resistant alleles, respectively. (DOCX) [file pone.0031582.s009.docx]

**Table S8.** Estimated dose-response values of segregating population of three different single pair inter strain crosses over multiple generations in *Tribolium castaneum*

| **Crosses** | **Discriminating Levels** | **Dose response (mg litre^-1^) over multiple generations** | | | |
| --- | --- | --- | --- | --- | --- |
| S-strain X Weak-R_1_ (SIC) |  | F_5_ | F_10_ | F_15_ | F_20_ |
|  | LC_10_ | 0.006 | 0.010 | 0.004 | 0.007 |
|  | LC_50_ | 0.008 | 0.013 | 0.008 | 0.010 |
|  | LC_90_ | 0.013 | 0.017 | 0.013 | 0.013 |
| S-strain X Strong-R (SIC) |  |  |  |  |  |
|  | LC_10_ | 0.005 | 0.013 | 0.005 | 0.006 |
|  | LC_50_ | 0.019 | 0.026 | 0.012 | 0.014 |
|  | LC_90_ | 0.070 | 0.055 | 0.034 | 0.047 |
| Weak-R_1_ X Strong-R (SIC) |  |  |  |  |  |
|  | LC_10_ | 0.020 | 0.023 | 0.017 | 0.017 |
|  | LC_50_ | 0.033 | 0.042 | 0.047 | 0.034 |
|  | LC_90_ | 0.098 | 0.090 | * | 0.083 |

* The lethal concentration to kill 90% of the population could not be estimated from the non- linear regression equation for this generation

SIC = Single Pair Inter strain cross
